# ZMYND8 suppresses *MAPT213* LncRNA transcription to promote neuronal differentiation

**DOI:** 10.1038/s41419-022-05212-x

**Published:** 2022-09-05

**Authors:** Santanu Adhikary, Vipin Singh, Ramesh Choudhari, Barbara Yang, Swagata Adhikari, Enrique I. Ramos, Soumi Chaudhuri, Siddhartha Roy, Shrikanth S. Gadad, Chandrima Das

**Affiliations:** 1 Biophysics and Structural Genomics Division, Saha Institute of Nuclear Physics, 1/AF Bidhannagar, Kolkata, 700064 India; 2 Structural Biology & Bio-Informatics Division, CSIR-Indian Institute of Chemical Biology, 4 Raja S. C. Mullick Road, Jadavpur, Kolkata, 700032 India; 3 Homi Bhaba National Institute, Mumbai, 400094 India; 4 Center of Emphasis in Cancer, Department of Molecular and Translational Medicine, Texas Tech University Health Sciences Center El Paso, 5001 El Paso Drive, El Paso, TX 79905 USA; 5 Mays Cancer Center, UT Health San Antonio MD Anderson Cancer Center, San Antonio, TX 78229 USA

**Keywords:** Mechanisms of disease, Long non-coding RNAs

## Abstract

Zinc Finger transcription factors are crucial in modulating various cellular processes, including differentiation. Chromatin reader Zinc Finger MYND (Myeloid, Nervy, and DEAF-1) type containing 8 (ZMYND8), an All-Trans Retinoic Acid (ATRA)-responsive gene, was previously shown to play a crucial role in promoting the expression of neuronal-lineage committed genes. Here, we report that ZMYND8 promotes neuronal differentiation by positively regulating canonical *MAPT* protein-coding gene isoform, a key player in the axonal development of neurons. Additionally, ZMYND8 modulates gene-isoform switching by epigenetically silencing key regulatory regions within the *MAPT* gene, thereby suppressing the expression of non-protein-coding isoforms such as *MAPT213*. Genetic deletion of *ZMYND8* led to an increase in the *MAPT213* that potentially suppressed the parental *MAPT* protein-coding transcript expression related to neuronal differentiation programs. In addition, ectopic expression of *MAPT213* led to repression of *MAPT* protein-coding transcript. Similarly, ZMYND8-driven transcription regulation was also observed in other neuronal differentiation-promoting genes. Collectively our results elucidate a novel mechanism of ZMYND8-dependent transcription regulation of different neuronal lineage committing genes, including *MAPT*, to promote neural differentiation.

## Introduction

Neural stem cells (NSCs) are multipotent precursors that differentiate into neuronal or glial lineage cells (astrocytes or oligodendrocytes) [1]. Stem cell niche microenvironment spatiotemporally regulates the differentiation of NSC to neurons via a process known as neurogenesis [2]. This is intriguingly a coordinated process involving the development of neurons, renewal of NSCs, specification of neural fate, migration, and maturation. Genes specific to neurogenesis are epigenetically controlled at target enhancers and promoters, and abrupt changes in the epigenetic landscape may lead to severe pathological conditions [3–5]. For example, rare, aggressive tumors such as pediatric high-grade gliomas (HGG) are associated with H3K27M (lysine-to-methionine substitution), resulting in a pathology-specific epigenetic state [6–8]. DNMTs and PRC2 have been previously reported to regulate neurogenesis [9]. Furthermore, chromatin remodellers such as MBD3/NuRD or REST/CoREST complex suppress neuronal differentiation by activating the pluripotency genes, including *NANOG* and *SOX2*, thus maintaining the undifferentiated state [10–12]. REST and CoREST regulate neural fate decisions via transcriptional and epigenetic mechanisms [13]. Collectively, these findings suggest a dynamic interplay between the transcription factors and epigenetic modifiers, which tightly control the differentiation process [14, 15].

ZMYND8 is a chromatin reader that recognizes dual histone modifications such as H3.1K36me2/ H4K16ac through its chromatin binding module, PWWP motif, Bromodomain, and PHD finger (PBP) and regulates All-Trans Retinoic acid (ATRA)- induced transcription programs [16]. ZMYND8, by its dual histone binding ability, induces a terminal differentiation program in breast cancer cells [17]. Other reports also suggest that it reads H3K4me1 and H3K14ac and suppresses prostate cancer metastasis [18, 19]. Recognition of H4K16ac by ZMYND8 recruits NuRD complex at DNA damage sites during DNA repair [20–22]. Moreover, ZMYND8, through its interaction with KDM5C, attenuates super-enhancer activation [23]. Transcriptionally, ZMYND8 can target oncogenes, multi-drug resistance genes, and stemness genes in breast cancer that are maintained in a poised epigenetic state and can repress them in association with KDM5C and EZH2 [24]. Overall, these studies depict the positive and negative role of ZMYND8 in transcription regulation [18, 25].

ZMYND8 interacts with Xenopus RCoR2 and Drebrin, a neuronal actin-binding protein, thereby regulating neuronal differentiation [26, 27]. *ZMYND8* has also been shown to be a critical gene that controls the anti-proliferative function of cancer cells mediated by ATRA [28]. In the present study, we show that a loss of ZMYND8 impairs neuronal differentiation from NSCs, without affecting other lineages. ZMYND8 directly regulates several differentiation-promoting genes through a distinct dual mechanism. Besides ZMYND8-dependent regulation of the target genes, it can also suppress differential isoform switching leading to alternative noncoding isoform expression. These noncoding transcripts synthesized from intra-gene promoters antagonize neuronal differentiation programs. As a proof of concept, a comprehensive study of ZMYND8-mediated *MAPT* gene transcription during neuronal differentiation has been performed here. We observed that the presence of ZMYND8 was key in positively regulating its full-length protein-coding gene transcription. At the same time, it suppressed the *MAPT213* non-coding isoform expression by recruiting different transcription repressor complexes. The absence of ZMYND8 promotes the expression of *MAPT213* ncRNA transcript, whose function is yet unknown. Furthermore, we demonstrate that the levels of full-length *MAPT* protein-coding transcripts and *MAPT213* non-coding transcript are different from each other. Notably, we show that the overexpression of *MAPT213* effectively suppresses the transcription of the full-length protein-coding *MAPT* gene. Collectively, our results demonstrate that ZMYND8-dependent unique mechanisms regulate the neuronal lineage committing genes to ensure a productive cell fate.

## Results

### ZMYND8 inhibits pluripotency and promotes neuronal differentiation

**Fig. 1:**
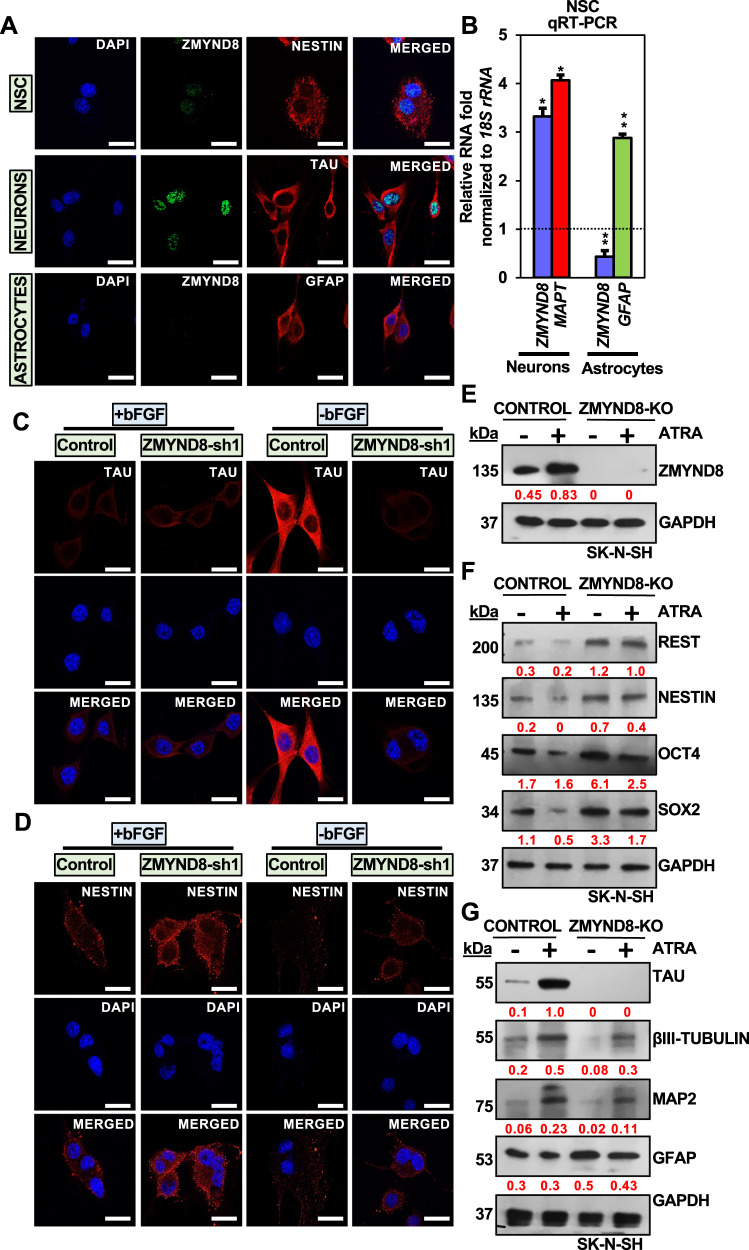
ZMYND8 regulates neuronal differentiation. **A**, **B** ZMYND8 expression in NSCs, neurons, and astrocytes by immunofluorescence (**A**) or qRT-PCR (**B**). NESTIN, TAU, and GFAP serve as pluripotency, neuronal and glial markers, respectively. The scale bar indicates 20 μm. **C**, **D** Immunofluorescence showing expression of TAU (neuronal) (**C**) or NESTIN (pluripotency) (**D**) in ZMYND8 knockdown-induced neurons differentiated from NSCs. The scale bar indicates 20 μm. **E**–**G** Immunoblot blot analysis showing expression of ZMYND8 (**E**), pluripotency marker (**F**), and neuronal differentiation marker (**G**) in *ZMYND8*-knockout (*ZMYND8*-KO) or control SK-N-SH cells with or without ATRA treatment. GAPDH serves as a loading control. The immunoblots are quantified and represented as numerical, reflecting the band intensity normalized to loading control. Error bars indicate sem.; *n* = 3; three independent experiments. A two-tailed *t*-test was used to calculate *P*-values. *P < 0.05; **P < 0.01.

ZMYND8 has been reported to regulate gene transcription [16, 25]. Previously, we have shown that ZMYND8 positively regulates the ATRA-induced neuronal gene expression through its chromatin reader function [16]. NSCs differentiate into neurons, astrocytes, or oligodendrocytes [1, 2]. We found that ZMYND8 is upregulated when NSCs differentiate into neurons and not into glial lineage cells (Fig. 1A, B). Short hairpin RNA (shRNA)-mediated silencing of *ZMYND8* in NSC (Supplementary Fig. 1A) induced the expression of pluripotency genes including *REST, POUSF1, NANOG, SOX2* and attenuated the expression of neuronal genes like *MAPT, TUBBIII, DRD2, SCN2A, LAMA1, NEUROG1* (Figs. 1C, D and Supplementary Fig. 1A). This was further confirmed by genetically deleting *ZMYND8* in SK-N-SH cells (Fig. 1E and Supplementary 1B, cell morphology shown in Supplementary Fig. 1C) or shRNA mediated silencing of *ZMYND8* in SH-SY5Y cells followed by ATRA treatment (Supplementary Fig. 1D), which triggered the neuronal differentiation, and inhibited the pluripotency genes (Figs. 1F, G, Supplementary 1B and E).

### ZMYND8 regulates ATRA-dependent transcriptional program

**Fig. 2:**
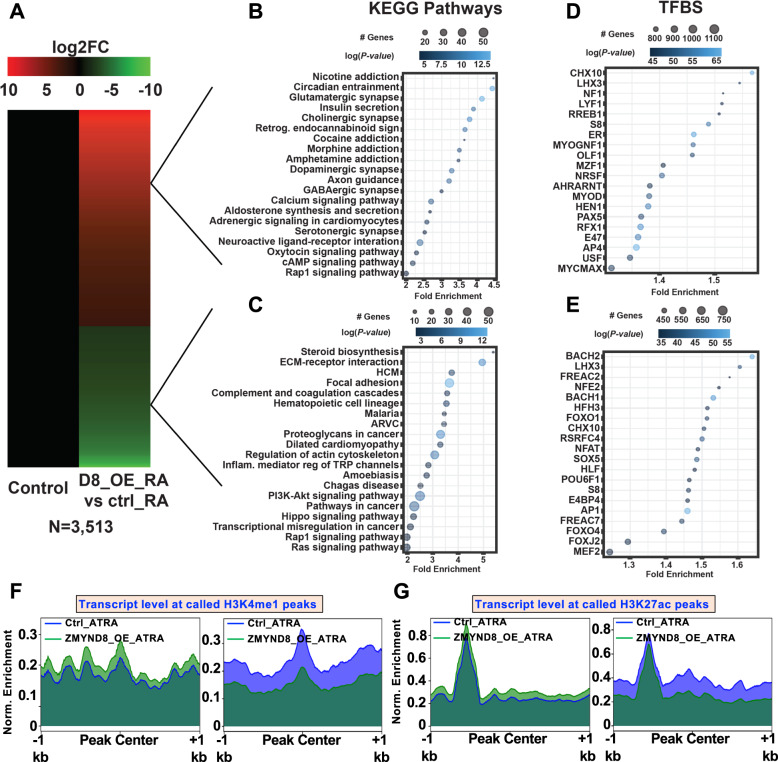
ZMYND8 overexpression along with ATRA treatment triggers key pathways involved in neuronal differentiation. **A** Heatmap showing differential gene expression upon ZMYND8 overexpression with ATRA treatment (annotated as ZMYND8_OE_ATRA). **B**–**E** KEGG pathways (**B**, **C**) and TFBS (**D**, **E**) of up (**B**, **D**) and down (**C**, **E**) regulated genes. **F**, **G** Transcript levels quantified at enhancers upon ATRA treatment with or without ZMYND8. Enhancers were defined by H3K4me1 and H3K27ac marks obtained from ENCODE ChIP-seq data in SK-N-SH cells.

**Fig. 3:**
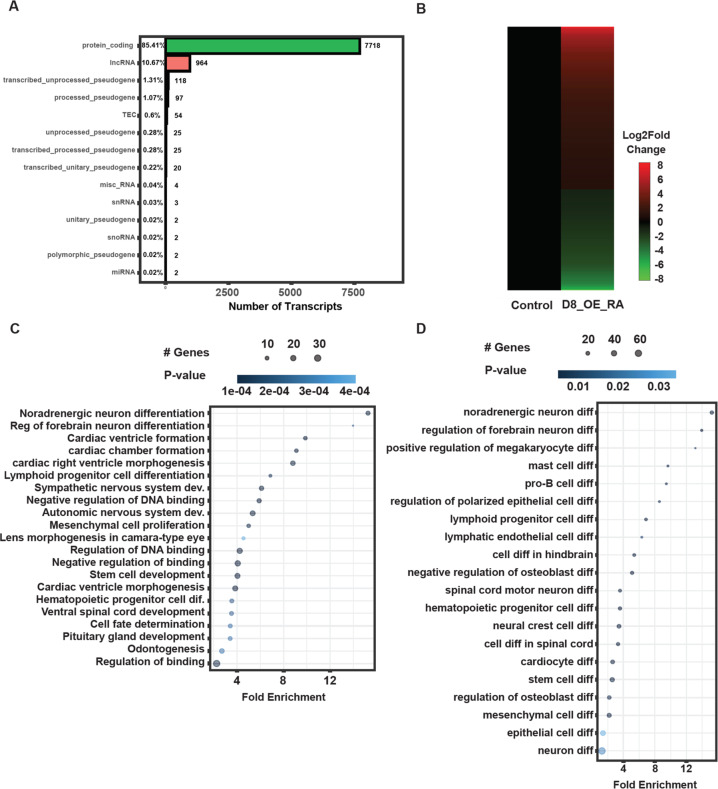
ZMYND8 regulates key pathways of neuronal differentiation. **A** Biotype-based classification of ZMYND8-regulate transcripts. **B** Heatmap depicting differential lncRNA gene expression upon ZMYND8 overexpression with ATRA treatment. **C** Genomic Regions Enrichment of Annotations Tool (GREAT) analysis of ZMYND-regulated lncRNAs. **D** The neuronal differentiation processes associated with ZMYND8-regulated lncRNAs as identified using GREAT.

To determine whether or not ZMYND8 localizes at transcriptionally active regions in the genome, we analyzed the publicly available genome-wide occupancy data for ZMYND8 and the factors that are associated with active transcription (H3K4me3, p300, H3K27ac, BRD4, and H3K4me1). Interestingly, we found that ZMYND8 colocalizes with transcription activators at transcriptionally active genomic regions (Supplementary Fig. 2), suggesting that ZMYND8 positively regulates transcription. Further, to interrogate its role in regulating primary ATRA-induced transcription, we performed a whole transcriptome analysis upon depleting ribosomal RNA in the presence of ectopically induced ZMYND8 upon ATRA treatment (Figs. 2 and 3). KEGG pathway analysis of differentially regulated genes showed that ZMYND8 modulates gene expression profiles associated with neuronal functions (Fig. 2A–C). Further, transcription factor target analysis of ZMYND8-regulated genes revealed that neuronal-specific transcription factors control the expression of these genes, such as *NRSF*, *MYOD*, *CHX10*, and *PRMT5* (Fig. 2D, E). Additionally, to investigate the role of ZMYND8 in regulating the transcription of active genomic regions, we analyzed the previously reported ChIP-seq data (from the ENCODE portal (https://www.encodeproject.org/) and visualized it on UCSC genome browser [29]) and identified the genomic regions in SK-N-SH cells that are enriched for H3K27ac and H3K4me1 (Supplementary Fig. 2). Using the total RNA-seq data, we quantified the levels of transcript synthesized from these regions. Interestingly, we found that ZMYND8 directly regulates the expression of these transcripts in response to ATRA treatment (Fig. 2F, G).

In addition, we sought to determine the RNAs regulated by ZMYND8, including noncoding RNAs. Biotype analysis of differentially regulated transcripts revealed that ZMYND8 also regulates the transcription of non-coding RNAs (Fig. 3A). The long noncoding RNAs (lncRNAs) were 10.67% of the differentially expressed transcripts regulated by ZMYND8. We subsequently characterized these transcripts further to delineate their roles in the biological processes of the ATRA differentiated cells (Fig. 3B–D). We used the “Genomic Regions Enrichment of Annotations Tool (GREAT)” to assess the biological significance of ZMYND8-regulated non-coding RNAs that are upregulated at least by one fold (Fig. 3B); intriguingly, we found that these RNAs are synthesized from the regions that are associated with neuronal differentiation (Fig. 3C). Additionally, GREAT analyses were used to identify all the differentiation processes may be associated with ZMYND8-upregulated lncRNAs (Fig. 3D). Collectively, these results suggest that ZMYND8 directly regulates transcription in response to ATRA treatment to control neuronal differentiation pathways.

### ZMYND8 regulates *MAPT* transcription through multiple mechanisms

**Fig. 4:**
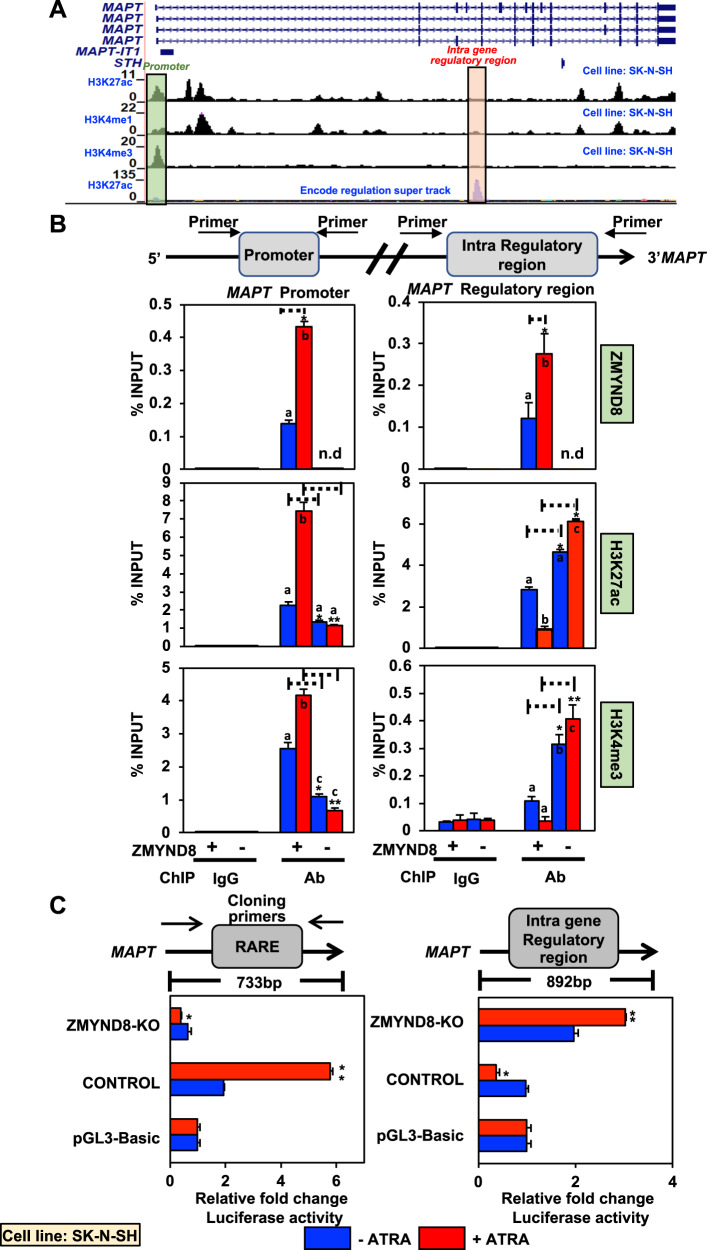
The absence of ZMYND8 shows increased transcription from *MAPT* intra-regulatory region. **A** UCSC Genome Browser track showing the histone modifications (H3K27ac and H3K4me3) at the *MAPT* gene; data was accessed from ENCODE. **B** Bar plots of qPCR showing enrichment of ZMYND8, H3K27ac, and H3K4me3 at the *MAPT* promoter (left panel) or intra-gene regulatory region (IGRR) (right panel) in ZMYND8-knockout (ZMYND8-KO) or control SK-N-SH cells with or without ATRA treatment. **C** Luciferase assay measuring the firefly luciferase activity of *MAPT* promoter (left panel) or intra-gene regulatory region (right panel) in *ZMYND8*-knockout (*ZMYND8*-KO) or control SK-N-SH cells with or without ATRA treatment. All firefly luciferase activities were normalized to renilla luciferase activity. Error bars indicate sem.; *n* = 3; three independent experiments. A two-tailed *t*-test was used to calculate *P*-values. *P < 0.05; **P < 0.01. One-way ANOVA was used to compute statistical differences. Boxes marked with a, b, and c are significantly different from each other padj-value = < 0.05.

Our previous study has shown that ZMYND8 regulates neuronal differentiation by controlling *TAU (MAPT)* expression [16]. ZMYND8 showed enhanced recruitment at the Retinoic Acid Response Element (RARE) site within *MAPT* promoter upon ATRA treatment, leading to its elevated expression [16]. Therefore we sought to elucidate the detailed mechanism of ZMYND8-dependent transcription of *MAPT*. A closer look at the *MAPT* gene through UCSC Genome Browser using ChIP-seq data in SK-N-SH cells showed that the promoter region has H3K27ac and H3K4me3 peaks (Fig. 4A). Interestingly, a clear H3K27ac peak was also observed at the intra-gene regulatory region (IGRR) (Fig. 4A). Furthermore, an enhanced H3K27ac occupancy was found in the absence of ZMYND8 followed by ATRA treatment in SK-N-SH and SH-SY5Y cells, as observed by ChIP assays (Fig. 4B and Supplementary Fig. 3A).

To assess the epigenetic landscape at the *MAPT* promoter (TSS) and the IGRR, we performed ChIP assays in *ZMYND8* knockout (*ZMYND8*-KO) SK-N-SH cells with or without ATRA treatment. ZMYND8 showed enhanced recruitment at the *MAPT* promoter and IGRR in an ATRA-dependent manner (Fig. 4B). Furthermore, we identified the domains involved in recruiting ZMYND8 at its target site. Interestingly we found that the MYND domain of ZMYND8, which is involved in mediating protein-protein interaction, is responsible for the recruitment at the *MAPT* promoter (Supplementary Fig. 3B). However, both the chromatin binding module PBP (PWWP-Bromo-PHD) and the MYND domain of ZMYND8 are crucial for its recruitment to the *MAPT* IGRR (Supplementary Fig. 3B).

Interestingly, ZMYND8 recruitment at the promoter was also accompanied by increased H3K27ac and H3K4me3 occupancy, which were drastically reduced in the *ZMYND8* knockout background (Fig. 4B). IGRR, on the other hand, showed a decrease in H3K27ac and H3K4me3 levels upon ATRA treatment, which got significantly elevated in the absence of ZMYND8 followed by ATRA treatment (Fig. 4B). However, no significant enrichment of H3K4me1 was found in the IGRR region in the lack of ZMYND8 even upon ATRA-treatment (Supplementary Fig. 3C), indicating that it is possibly a bona fide promoter of *MAPT* isoform, not an enhancer. Additionally, several other neuronal genes, including *DRD2* and *SCN2A*, showed similar H3K27ac peaks within the gene body region, further validated by ChIP assays (Supplementary Fig. 3D-3G). Therefore, our results indicated a repressed epigenome at the *MAPT* promoter in the absence of ZMYND8, thereby suppressing *MAPT* transcription. Contrastingly, the IGRR region was transcriptionally triggered in the absence of ZMYND8. Our results could be extrapolated to other neuronal genes harboring the intra-regulatory region.

To determine the outcome of these alterations in the epigenome, we assessed the *MAPT* promoter activity in the absence of ZMYND8. The *MAPT* RARE site was cloned in a pGL3 basic vector. Increased firefly luciferase activity was observed upon ATRA treatment exclusively in the presence of ZMYND8 (Fig. 4C). However, the loss of ZMYND8 could not activate the *MAPT* promoter upon ATRA treatment (Fig. 4C). Furthermore, to elucidate the transcriptional activity of the IGRR, it was cloned in the pGL3 basic vector, and luciferase activity was scored. Interestingly the IGRR was transcriptionally active only in the absence of ZMYND8 upon ATRA treatment in SK-N-SH cells (Fig. 4C). Similarly, *ZMYND8* knockdown attenuated the promoter activation and augmented IGRR firing in SH-SY5Y cells upon ATRA treatment (Supplementary Fig. 3H and 3I). These results indicate that ZMYND8 differentially regulates transcription from the promoter, and IGRR, which is similar across different neuronal cell lines.

### ZMYND8 regulates the epigenetic landscape of the *MAPT* gene

**Fig. 5:**
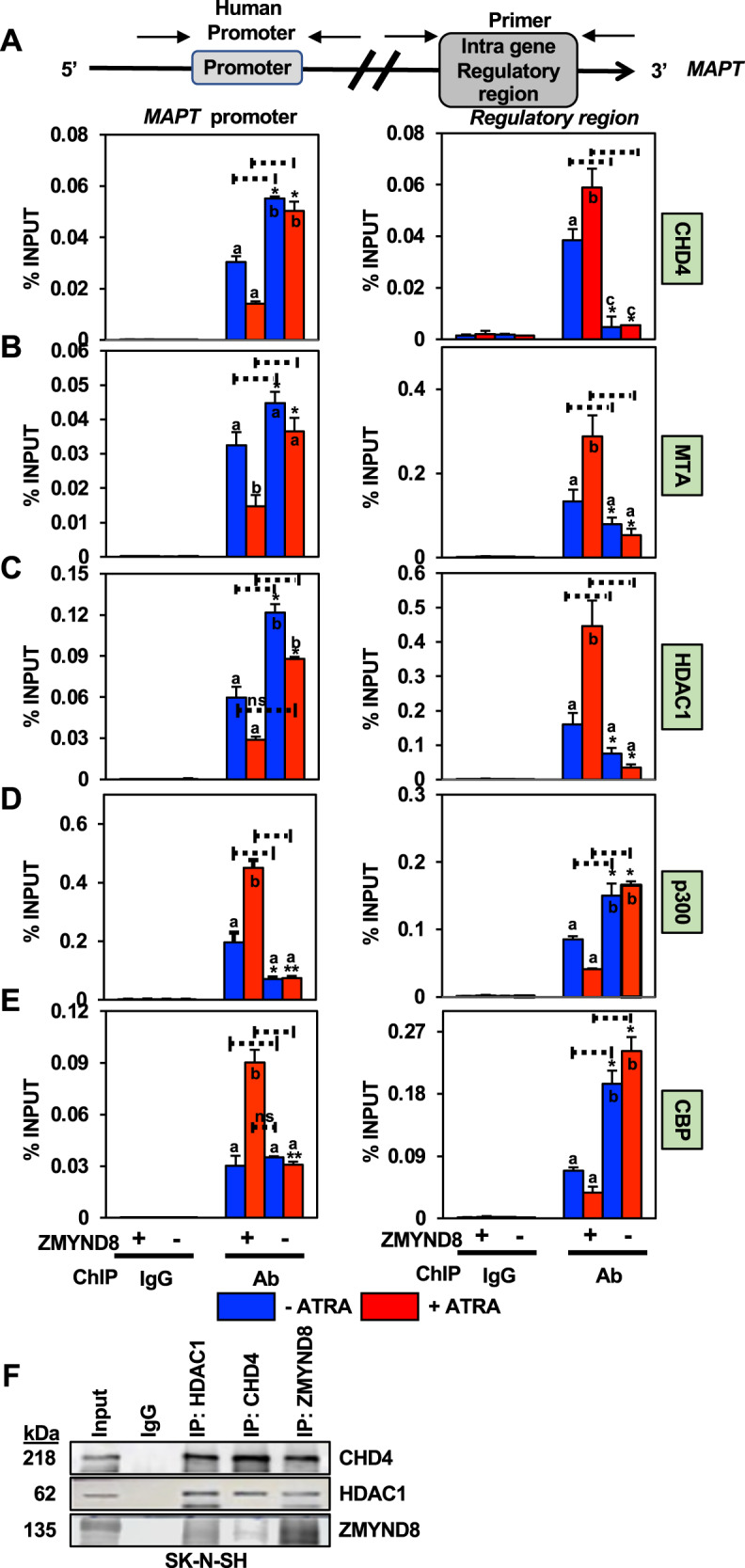
ZMYND8 is associated with the transcription repressor complex at the *MAPT* intra-regulatory region. **A**–**E** Bar plots of qPCR showing enrichment of CHD4 (**A**), MTA1 (**B**), HDAC1 (**C**), p300 (**D**), and CBP (**E**) at the *MAPT* promoter and IGRR in *ZMYND8*-knockout (*ZMYND8*-KO) or control SK-N-SH cells with or without ATRA treatment. Error bars indicate sem.; *n* = 3; three independent experiments. A two-tailed *t*-test was used to calculate *P*-values. *P < 0.05; **P < 0.01. One-way ANOVA was used to compute statistical differences. Boxes marked with a, b, and c are significantly different from each other padj-value = <0.05. **F** Coimmunoprecipitation showing ZMYND8 associates with NuRD subunits (HDAC1 and CHD4) and vice versa.

To assess the epigenetic landscape of the *MAPT* promoter and the IGRR site, a series of ChIP assays were performed upon ATRA treatment in *ZMYND8*-KO SK-N-SH cells. Loss of ZMYND8 led to an increase in the recruitment of NuRD complex (CHD4, MTA1, and HDAC1) at the promoter region (Fig. 5A–C), indicating NuRD independent role of ZMYDN8. The absence of ZMYND8 also decreased p300 and CBP recruitment at the promoter region (Fig. 5D, E). Next, we wanted to check whether enrichment of ZMYND8 at the IGRR site was through its NuRD-dependent function. Indeed, removal of CHD4, MTA1, and HDAC1 (NuRD complex) from the IGRR was found upon loss of ZMYND8 in ATRA-treated condition (Fig. 5A–C). Contrastingly, increased occupancy of CBP and p300 was observed at the IGRR upon ZMYND8 loss, followed by ATRA treatment (Fig. 5D, E). Moreover, coimmunoprecipitation with NuRD component subunits (CHD4 or HDAC1) showed a strong interaction with ZMYND8 in the presence of ATRA (Fig. 5F). Reciprocal pull down of ZMYND8 also showed its association with NuRD complex (Fig. 5F).

**Fig. 6:**
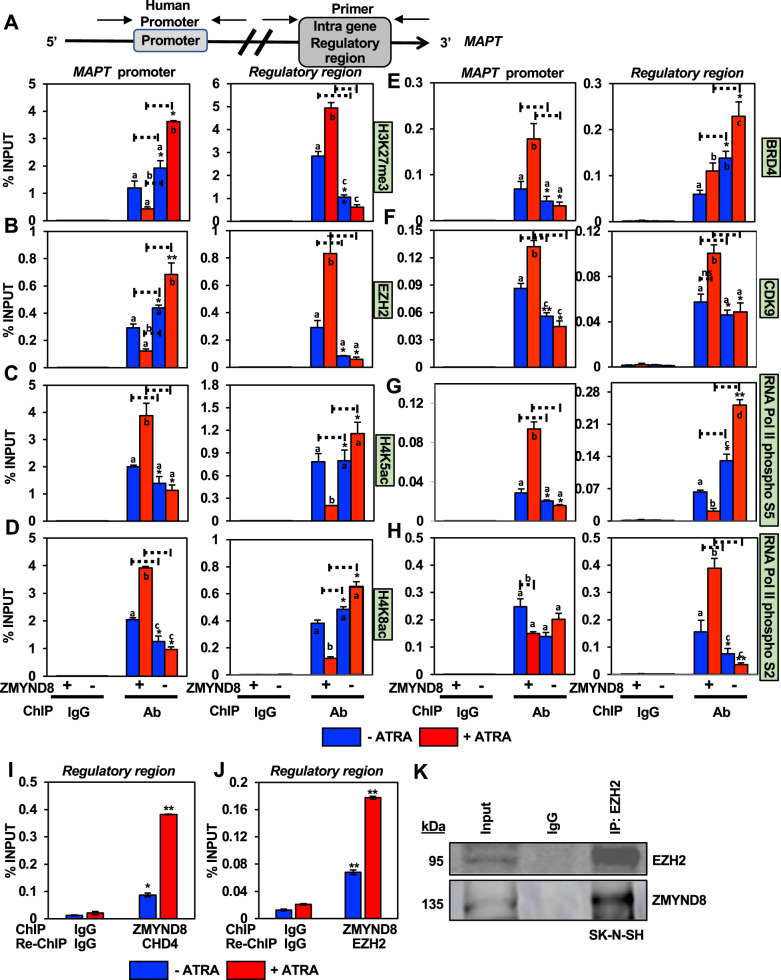
ZMYND8 switches to the transcription activating epigenetic landscape at the intra-gene regulatory region of *MAPT*. **A**–**H** Bar plots of qPCR showing enrichment of H3K27me3 (**A**), EZH2 (**B**), H4K5ac (**C**), H4K8ac (**D**), BRD4 (**E**), CDK9 (**F**), RNA pol II phosphoS5 (**G**), and RNA pol II phosphoS2 (**H**) at the *MAPT* promoter and IGRR in *ZMYND8*-knockout (*ZMYND8*-KO) or control SK-N-SH cells with or without ATRA treatment. **I**, **J** Re-ChIP assays show ZMYND8 forms complex with CHD4 (subunit of NuRD) (**I**) and EZH2 (subunit of PRC2) (**J**) at the IGRR site. Error bars indicate sem.; *n* = 3; three independent experiments. A two-tailed *t*-test was used to calculate *P*-values. *P < 0.05; **P < 0.01. One-way ANOVA was used to compute statistical differences. Boxes marked with a, b, and c are significantly different from each other padj-value = < 0.05. **K** Coimmunoprecipitation shows an association between EZH2 and ZMYND8.

Increased recruitment of EZH2 explains the increased H3K27me3 levels at the promoter in the *ZMYND8*-KO cells followed by ATRA treatment (Fig. 6A, B). In contrast, a significant decrease in occupancy of EZH2 and H3K27me3 levels was found at the IGRR site in *ZMYND8*-KO cells (Fig. 6A, B). Loss of well-known transcription activation marks such as H4K5ac and H4K8ac was observed from TSS in the absence of ZMYND8 (Fig. 6C, D). However, increased occupancy of H4K5ac or H4K8ac at the *MAPT* IGRR was found in *ZMYND8*-KO cells followed by ATRA treatment, indicating an increase in transcription activating signatures (Fig. 6C, D). The absence of ZMYND8 also decreased BRD4, CDK9, and RNA polymerase II phosphoS5 from the promoter region (Fig. 6E–G). However, there was no alteration in the levels of RNA polymerase II phosphoS2 in this region (Fig. 6H). Contrastingly, an increase in BRD4 and a decrease in CDK9 levels were observed at IGRR upon loss of ZMYND8, followed by ATRA treatment (Fig. 6E, F). Surprisingly, an enhanced occupancy of RNA polymerase II phosphoS5 followed by a decrease in RNA polymerase II phosphoS2 upon ZMYND8 loss indicated a de novo transcription event from the IGRR of *MAPT* (Fig. 6G, H). Similar observations could be noted at the *MAPT* promoter and IGRR upon *ZMYND8* knockdown in SH-SY5Y cells (Supplementary Fig. 4). To delineate that ZMYND8 is associated with NuRD complex and EZH2 (as a component of PRC2) at the IGRR site, we performed two separate Re-ChIP assays. Upon immunoprecipitating ZMYND8 followed by CHD4, we observed a ZMYND8/CHD4 complex recruitment at IGRR as observed by Re-ChIP assay (Fig. 6I). Similar complexes of ZMYND8/EZH2 could also be seen at the IGRR site (Fig. 6J). Since we had already observed that ZMYND8 and NuRD sub-units interact with each other, we hypothesized the assembly of the ternary complex was through the direct association of ZMYND8 with EZH2. And we observed a ZMYND8/EZH2 complex formation by immunoprecipitation assays (Fig. 6K). These results suggest that ZMYND8 positively regulates MAPT transcription by establishing a transcriptionally favorable epigenetic environment at the *MAPT* promoter. However, loss of ZMYND8, on the other hand, is critical in initiating transcription from IGRR, which generates a noncoding transcript annotated as *MAPT213* (noncoding isoform) in ENCODE.

### ZMYND8 loss promotes the synthesis of a transcript from IGRR of the *MAPT* gene

**Fig. 7:**
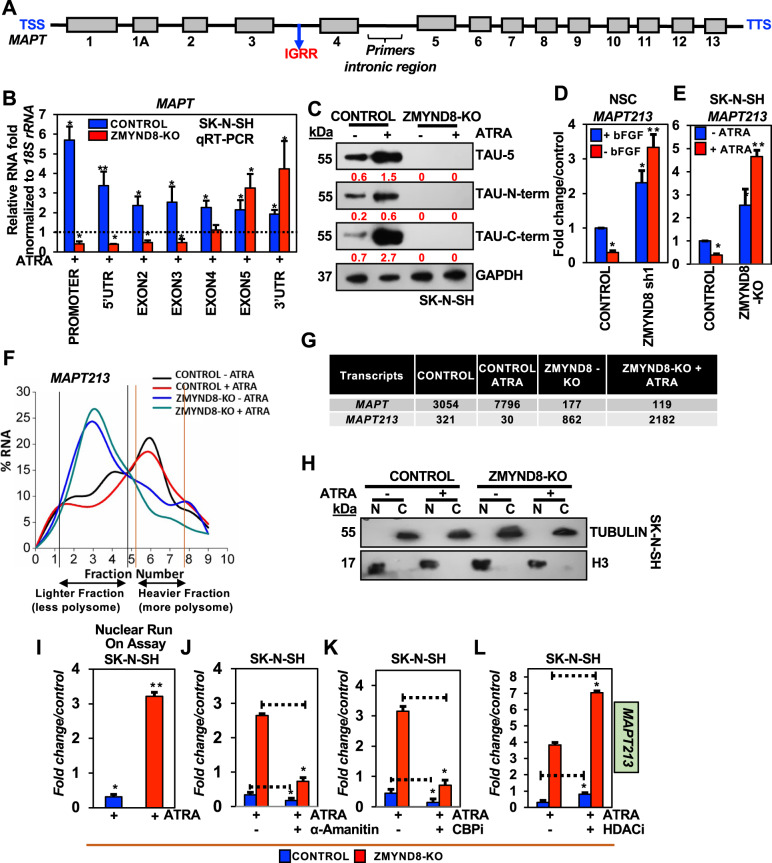
The absence of ZMYND8 triggers the expression of the non-coding transcript from the intra-gene regulatory region of the *MAPT* gene. **A** Schematic representation of *MAPT* gene representing the TSS (Transcription Start Site), exons (1-13), intra-gene regulatory region, an intronic region from where primers were designed for the noncoding transcript, and TTS (Transcription Termination Site). **B** qRT-PCR showing expression of *MAPT* transcripts from the promoter, 5'UTR, Exons 2–5, and 3'UTR in *ZMYND8*-knockout (*ZMYND8*-KO) or control SK-N-SH cells with or without ATRA treatment. **C** Immunoblots showing TAU expression with anti-TAU antibody against epitopes specific to N-term, C-term, or TAU5 in *ZMYND8*-knockout (*ZMYND8*-KO) or control SK-N-SH cells with or without ATRA treatment. GAPDH served as a loading control. The immunoblots are quantified and represented as numerical, reflecting the band intensity normalized to loading control. **D**, **E** qRT-PCR showing expression of *MAPT Intra gene transcript (MAPT213)* upon *ZMYND8* knockdown in bFGF depleted NSC (**D**), or ATRA treated *ZMYND8* knockout (*ZMYND8*-KO) SK-N-SH cells (**E**). **F** Polysome profiling to determine the polysome bound fractions of *MAPT Intra gene transcript (MAPT213)* in *ZMYND8*-knockout (*ZMYND8*-KO) or control SK-N-SH cells with or without ATRA treatment. **G** Copy number /number of molecules of full-length *MAPT* and *MAPT Intra gene transcript (MAPT213)* determined from *ZMYND8*-knockout (*ZMYND8*-KO) or control SK-N-SH cells with or without ATRA treatment. **H** Immunoblot showing the efficiency of cell fractionation. Histone H3 and Tubulin are used as nuclear or cytosolic markers, respectively. **I** Nuclear run-on assay to show *MAPT Intra gene transcript* transcription efficiency in *ZMYND8*-knockout (*ZMYND8*-KO) or control SK-N-SH cells with or without ATRA treatment. **J**–**L** qRT-PCR showing expression *of MAPT Intra gene transcript* under α-Amanitin (10 μg/ml for 16 h) (**J**), CBP inhibitor (Curcumin 10 μM for 16 h) (**K**), or HDAC1 inhibitor treatment (TSA 2 μM for 24 h) (**L**) in *ZMYND8*-knockout (*ZMYND8*-KO) or control SK-N-SH cells with or without ATRA treatment. Error bars indicate sem.; *n* = 3; three independent experiments. A two-tailed *t*-test was used to calculate *P*-values. *P < 0.05; **P < 0.01.

Loss of ZMYND8 promotes the transcription from *MAPT* IGRR (*MAPT213*) instead of the full-length *MAPT*. Processed *MAPT* full-length transcript has 13 exons (Fig. 7A), and there are 6 major alternatively spliced isoforms. We analyzed the different *MAPT* exon-specific transcripts. Remarkably, the absence of ZMYND8 led to downregulation of *MAPT* transcripts (exon 1–3) and an increase in the expression of exon 4 onwards upon ATRA treatment SK-N-SH cells (Fig. 7B). Similar results were observed in *ZMYND8*-knockdown SH-SY5Y cells followed by ATRA treatment (Supplementary Fig. 5A). Furthermore, we analyzed the expression of MAPT protein, TAU, using three separate antibodies against N-term, C-term, or TAU5. Immunoblot analysis using all the antibodies showed no TAU protein production in *ZMYND8*-KO SK-N-SH cells with or without ATRA treatment (Fig. 7C). This suggests the differential expression of *MAPT* isoform lacks coding exons (1–3). Next, we wanted to delineate whether this transcript is non-coding, for which we designed primers encompassing the intronic region between exons 4 and 5. The high levels of this non-coding transcript (ncRNA) were found in differentiated neurons from NSCs, and ATRA-treated SK-N-SH and SH-SY5Y cells in the absence of ZMYND8 (Fig. 7D, E and Supplementary Fig. 5B). Interrogation of the GENCODE database showed that *MAPT* ncRNA corresponds to a *MAPT* isoform known as *MAPT213*. To analyze the coding ability of *MAPT213*, polysome profiling of control or *ZMYND8*-KO ATRA treated SK-N-SH cells was performed. Fewer polysomes were bound to the *MAPT213* (Fig. 7F). Furthermore, we quantitated the copy number of *MAPT* and *MAPT213* in *ZMYND8*-KO ATRA treated SK-N-SH cells. An inverse correlation was found between *MAPT* and *MAPT213* copy number per cell in control or *ZMYND8*-KO ATRA treated SK-N-SH cells (Fig. 7G). These results indicate gene isoform switching and the synthesis of ncRNA within the *MAPT* gene upon loss of ZMYND8.

To functionally characterize, we determined the subcellular localization of the *MAPT213*. First, the fractionation efficiency was verified by immunoblotting with cytosol-specific marker, Tubulin or nucleus-specific marker, histone H3 (Supplementary Fig. 5C). An increased *MAPT213* level in the nuclear fraction was found upon ATRA treatment in *ZMYND8*-KO SK-N-SH cells (Supplementary Fig. 5D). The cytosolic fraction was normalized to *18* *S rRNA* and the nuclear fraction to *U6snRNA*. Also, we performed nuclear run-on assay in control or *ZMYND8*-KO SK-N-SH cells to assess the transcription efficiency of *MAPT213*. The cellular fractionation was verified by immunoblotting with specific cytosolic (Tubulin) and nuclear (H3) markers (Fig. 7H). The nuclear run-on assay showed a higher transcription efficiency of *MAPT213* in *ZMYND8*-KO SK-N-SH cells with ATRA treatment (Fig. 7I). Also, treatment with α-Amanitin showed inhibition of RNA Polymerase II, resulting in the decrease of *MAPT213* levels, indicating an RNA polymerase II-dependent transcription (Fig. 7J). Next, we wanted to decipher the role of epigenetic modulators that could impact the expression of this non-coding transcript. To this end, ATRA-stimulated control and *ZMYND8*-KO SK-N-SH cells were treated with CBP inhibitor (curcumin, 10 μM for 16 h) and HDAC inhibitor {Trichostatin A (TSA), 2 μM for 24 h}. Treatment with curcumin led to repression, whereas TSA treatment led to upregulation of the *MAPT213* in these cells (Fig. 7K, L). Remarkably, analysis of RNA-seq data extracted from GTEx brain samples showed a correlation between *ZMYND8* and *MAPT213* expression was low, as compared to ZMYND8 and full-length *MAPT* (Supplementary Fig. 5E). Moreover, in a manner similar to the majority of the noncoding RNAs, *MAPT213* is primate-specific (Supplementary Fig. 5F) and not conserved in mice.

**Fig. 8:**
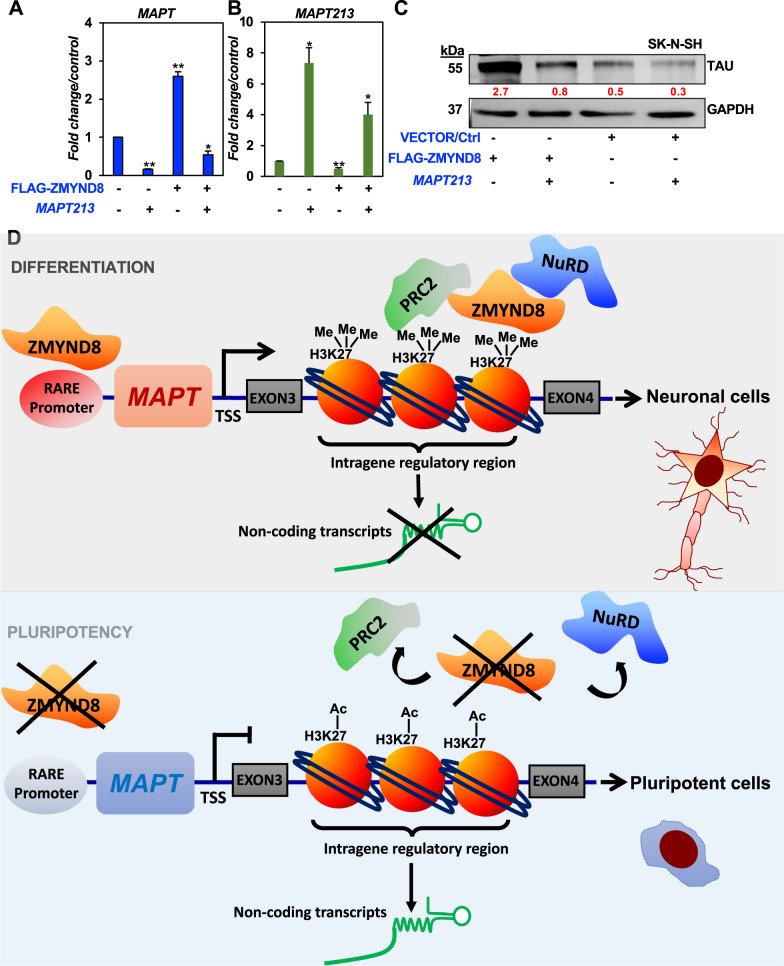
Antagonistic relationship between *MAPT213* and full-length *MAPT* transcription. **A**, **B** qRT-PCR showing expression of full-length *MAPT* (*MAPT*) (**A**) or *MAPT213* (*Intra gene transcript*) (**B**) in either *ZMYND8* alone, *MAPT213* alone, or both *ZMYND8* and *MAPT213* overexpressed SK-N-SH cells. **C** Immunoblots showing expression of TAU in either *ZMYND8* alone, *MAPT213* alone, or both ZMYND8 and *MAPT213* overexpressed SK-N-SH cells; GAPDH served as a loading control. The immunoblots are quantified and represented as numerical, reflecting the band intensity normalized to loading control. **D** Model depicting ZMYND8-dependent transcriptional regulation of *MAPT*. Error bars indicate sem.; *n* = 3; three independent experiments. A two-tailed *t*-test was used to calculate *P*-values. *P < 0.05; **P < 0.01.

Further, to understand whether or not *MAPT213* has any functional role in regulating its host *MAPT* gene expression, the *MAPT213* transcript was cloned and expressed alone or in combination with *ZMYND8* in SK-N-SH cells. Although the expression of full-length *MAPT* transcript was induced by ZMYND8 overexpression, it gets repressed by ectopic expression of *MAPT213* (Fig. 8A, B). Remarkably, the effect of *MAPT213* overexpression on MAPT wild-type transcription was dominant negative as even ZMYND8 overexpression could not alleviate the repressive effect (Fig. 8B). Similar results could also be observed in the MAPT protein level (Fig. 8C). Collectively, our results indicate a gene isoform switching of *MAPT* gene resulting in expression of *MAPT213* that lacks protein-coding ability. *MAPT213*, in turn, represses the full-length MAPT, thus impairing neuronal differentiation.

## Discussion

Differentiation of NSCs to neurons or glial lineage is well-orchestrated by epigenetic programs (which include histone PTMs and ncRNA mediated regulations) and transcription factor dynamics [3, 4]. Recently, zinc finger family protein ZMYND8 has been identified as a chromatin reader regulating ATRA-dependent transcription [16].

The role of *ZMYND8* or mutated *ZMYND8* is not known in neurological disorders. However, it has been shown to interact with Drebrin ADF-H, a protein that has been demonstrated to be associated with Alzheimer’s disease [26]. These studies led us to determine the function of *ZMYND8* in neuronal differentiation. Here we show that ZMYND8 plays a crucial role in the fate determination of the NSCs to the neuronal lineage. ZMYND8 loss inhibits neural differentiation and maintains the pluripotency state of the cells, evident from upregulated expression of pluripotency genes, *NESTIN* and *SOX2*, and attenuated levels of lineage commitment genes such as *MAPT* or *TUBBIII*. The KEGG pathway analysis indicated that ZMYND8 regulates pathways associated with neuronal functions suggesting a substantial role of ZMYND8 in ATRA-dependent transcription and neuronal differentiation. Further investigation is required to identify the in-depth role of ZMYND8 in the later stages of neuronal differentiation and the functioning of neurons.

Interestingly, apart from differentially regulating the protein-coding transcripts, we observed that ZMYND8 also regulates the noncoding transcripts. The previous report has identified that the *Trojan* lncRNA is instrumental in degrading ZMYND8, thereby promoting Triple-Negative Breast Cancer (TNBC) progression [30]. However, to date, there is no detailed study to identify the role of ZMYND8 in regulating noncoding transcription during neuronal differentiation. Interestingly, we observed that many of these noncoding transcripts regulated by ZMYND8 were intragenic with hitherto unreported functions. Therefore, the detailed function of these non-coding transcripts needs to be investigated to unravel the different mechanisms involved in neurogenesis.

Non-coding RNAs regulate gene expression at several levels via modulating chromatin structure and function, thereby regulating local and distal genes [31]. Long non-coding RNAs (lncRNAs) such as *ANRIL* facilitate the recruitment of PRC2 at *CDKN2A* and *CDKN2B* promoters to repress transcription and regulate cellular senescence [32]. LncRNAs also inhibit the recruitment of transcription factors or RNA polymerase II [33], thus altering histone modifications to reduce chromatin accessibility [34]. Enhancer-derived lncRNAs modulate chromatin topology associated with chromatin regulators to activate gene expression [35]. We were interested in comprehending the mechanism by which ZMYND8 regulates noncoding RNAs in the context of neuronal differentiation. Previously we observed that ZMYND8 remarkably alters the transcription of several neuronal differentiation markers, including *MAPT*, upon ATRA-treatment in neuroblastoma cells [16].

Interestingly, we had attributed the same in two different contexts, (i) a promoter-specific regulatory mechanism, where ZMYND8 forms complex with RNA Polymerase II initiation competent complex, and (ii) a regulatory mechanism specific to gene body, where ZMYND8 could get recruited to the modifications such as H3.1K36me2/H4K16ac via its chromatin reader function and possibly preventing the cryptic transcription events. However, a detailed study on the latter mechanism is lacking. Interestingly, microtubule-associated protein TAU or *MAPT* is an early neuronal differentiation marker expressed in the axonal region. The functional significance of different TAU isoforms is comprehended through their regulation during development. Finally, we investigated the mode of regulation of the *MAPT* gene by ZMYND8 during ATRA-induced neuronal differentiation.

In the human brain, six different TAU isoforms are synthesized by alternative splicing or gene-isoform switching of *MAPT* [36]. These isoforms vary with either none, one or two N-terminal inserts, and at C-terminus with either three or four microtubule-binding domains. However, mining *MAPT* gene transcripts in Ensembl clearly shows the existence of thirteen different protein-coding isoforms and one isoform that shows nonsense-mediated decay. The function of all of these transcripts is not well characterized. Four other isoforms do not code for any protein, either processed transcripts or retained intron. *MAPT213* is one such non-coding transcript whose function remains to be elucidated. We observed that transcription of *MAPT* full length and *MAPT213* follows distinct mechanisms steered by ZMYND8. The cis-transcriptional regulatory mechanism by long noncoding RNA is well-known. However, lncRNAs such as *Dali* regulate neuronal differentiation at local and distal genomic loci [37]. We observed that ZMYND8 represses transcription of *MAPT213*, which inhibits the *MAPT* full-length transcription upon activation. However, whether *MAPT213* has similar distal regulatory mechanisms is yet to be identified.

We finally aimed to understand the molecular details of ZMYND8-mediated transcription regulation of *MAPT* full length and *MAPT213*. ZMYND8 has been involved in both activation and repression of gene transcription. ZMYND8 associates with CHD4, a key component of the NuRD complex via its MYND domain, thereby repressing the gene transcription [22] at the DNA damage site. Similarly, its interaction with JARID1D or KDM5C suppresses the activation of metastasis-linked genes in various cancers [38]. Furthermore, ZMYND8 interacts with RNA pol II phosphorylated at S5 at the promoter-proximal region and activates ATRA-induced gene transcription [16].

Interestingly, the intra-gene regulatory transcription event from within the *MAPT* gene peeked in the absence of ZMYND8, as observed through the predominance of epigenetic signatures of H3K27ac and H3K4me3. Further, there was differential recruitment of transcription regulatory complexes at these two sites of the *MAPT* gene depending upon the presence of ZMYND8. When ZMYND8 was present, there was preferential recruitment of coactivators p300/CBP at the *MAPT* promoter, which was further induced upon ATRA treatment. However, in the absence of ZMYND8, a preferential gene isoform switching mechanism was initiated, which led to the production of the intra-gene transcript, *MAPT213*, upon removal of NuRD repressor complex and recruitment of p300/CBP. The alleviation of transcription repression followed by activation events could also be observed by favorable recruitment of transcription complex subunits BRD4/RNA Pol II phosphorylated at S5.

Collectively our results showed the dual function of ZMYND8 in promoting neuronal differentiation (Fig. 7D). Its interaction with the RNA-polymerase II complex transcriptionally activates several neuronal genes, including *MAPT*. Through association with transcription repressor complexes such as NuRD or PRC2, it represses the intra-gene transcription. In addition, it inhibits the synthesis of *MAPT213*, promoting the processive transcription of key neuronal gene *MAPT* involved in neural differentiation. This dual mode of transcription regulation of ZMYND8 is validated in multiple candidate genes regulating neuronal differentiation programs and could be considered a generalized mechanism. Suppressing the intra-genic transcription and promoting the neuronal genes’ full-length transcription events could have broader implications for cancer prevention. Thus, ZMYND8 could be employed to program the neuroblastoma cells towards a differentiated phenotype and has a huge therapeutic potential.

## Methods

### Cell lines, cell culture, and transfections

HEK293T, SK-N-SH, and SH-SY5Y cells were cultured in DMEM (Dulbecco’s Modified Eagle’s Medium; Gibco). The media was supplemented with 10% FBS (fetal bovine serum; Gibco) and 1% antibiotic-antimycotic (Gibco), and cells were maintained at humified37 °C and 5% CO_2_. A negative test report of mycoplasma contamination was obtained for all the cell lines used in this study. DNA fingerprinting was used to validate all the cell lines used in the study at MD Anderson Cancer Center Characterized Cell line core facility. Transient transfections were performed using Lipofectamine 2000 (Invitrogen) in cells counted and seeded in 12-well or 6-well, or 6-cm dishes per the manufacturer’s protocol.

### Cell differentiation

Neural Stem Cells were obtained from Gibco, Invitrogen and were maintained in Knockout DMEM/F12 supplemented with 2% StemPRO NSC SFM, EGF (20 ng/mL), bFGF (20 ng/mL), and 2 mM Glutamax. In the presence of a basic fibroblast growth factor (bFGF), the neural stem cells remain undifferentiated, and withdrawal of bFGF leads to its differentiation into the neuronal lineage [39]. The cells were maintained in dishes coated with CELLstart CTS. For neuronal differentiation, the cells were seeded in dishes coated with poly-L-ornithine and Laminin. The following day the cells were shifted to neuronal differentiation medium (Neurobasal medium, supplemented with B27 and Glutamax) and kept for 7 days; every 2 days, the medium was changed. For astrocyte differentiation, the cells were seeded on geltrex-coated plates. The following day the cells were replenished with the astrocyte differentiation medium (DMEM supplemented with N-2, GlutaMAX-I, and FBS) and kept for 7 days; a fresh medium was given every alternative 2 days.

SK-N-SH and SH-SY5Y cells were differentiated into neurons by shifting the cells in a differentiation medium (DMEM/F12 with 5% FBS and 1% Antibiotic-antimycotic) and supplemented with 10 μM ATRA. Every alternate day, cells were replenished with a fresh medium containing ATRA.

### ZMYND8 knockout cell generation

For stable *ZMYND8* knockout cell generation, the protocol was described elsewhere [40]. Briefly, the gRNAs were cloned into the pX459 vector. The plasmid was transfected in the target cell using lipofectamine 2000 as per the manufacturer’s protocol and subject to puromycin selection (5 μg/ml for 48 h). Stable knockout of ZMYND8 was confirmed through western blotting using the ZMYND8 antibody.

### RNA interference via lentiviral production

shRNA plasmids targeting *ZMYND8* in pLKO.1-puro backbone (from oriGene) was screened for the best knockdown. Lentivirus-mediated knockdown was carried out as described earlier [41]. Briefly, HEK293T cells were seeded in 10-cm dishes at a density of 3 × 10^5^ cells. 14 μg of shRNA, 7 μg of pPAX2 (packaging vector), and 3.5 μg of pMD2.G (envelope vector) were transfected in combination with the amount mentioned above using lipofectamine 2000 into the cells. 48 h of post-transfection, the conditioned medium having viral particles was collected and filtered through a 0.45 μm filter. Transduction was performed on target cells by adding viral sup and fresh media (1:1) with 10 μg/ml polybrene solution (Sigma) for four rounds with an interval of 4 h. The cells that got transduced were selected stably using puromycin (Sigma) (3 μg/ml for SH-SY5Y and 1 μg/ml for NSC) for 3 days.

### Overexpression via lentiviral production

ZMYND8 cloned into pInducer20 vector was transfected with pMD2G and psPAX2 plasmids into HEK293T cells (ATCC, CRL-3216) using Lipofectamine 3000 kit (Thermo Fisher Scientific, L3000015) to generate lentiviruses. Culture medium from transfected 293T cells was collected at 48 h and 72 h post-transfection and filtered through a 0.45 μm filter to transduce SK-N-SH cells, with the addition of polybrene (0.5 μg/ml). Stably transduced cells were then selected by Geneticin^TM^ (G418) at 800 μg/ml(Thermo Fisher Scientific, 11811031). Cell seeding densities were at 2 × 10^6^ cells in a 10 cm dish for 293 T and 1 × 10^6^ cells in a 10 cm dish for SK-N-SH. Cells were transfected/transduced 24 h after plating.

### Cloning of *MAPT213* ncRNA

Genomic DNA isolated from SK-N-SH cells (ATCC, HTB-11) using PureLink^TM^ Genomic DNA Mini Kit (Invitrogen, K1820-01) was used as a template to amplify *MAPT-213*, a single-exon transcript, by PCR method. PCR reaction was performed using Phusion DNA polymerase (New England Biolabs Inc., M0530L). PCR product was visualized with 1% SYBR-stained agarose gel, and the band at the correct size was excised and purified. Digested gel-purified *MAPT-213* inserts with KpnI and EcoRV restriction sites were cloned into the pcDNA3.0 vector. *MAPT-213* plasmid was scaled up in DH5α competent cells (Invitrogen, 18265017) and extracted with PureLinkHiPure Plasmid Filter Maxiprep Kit (Invitrogen, K210017). *MAPT213* sequence was confirmed with Sanger sequencing.

### Quantitative real-time PCR (qRT-PCR)

The isolation of total cellular RNA was performed by the TRIzol method. For reverse transcription of total cellular RNA to complementary DNA (cDNA), as per the manufacturer’s protocol, Revertaid Fast strand cDNA synthesis kit (Thermo Scientific) was used. Briefly, the total RNA, Reverse Transcriptase, and random hexamer or Oligo dT primer (100 pmol) were used for reverse transcription. SYBR GREEN mix from ABI (Applied Biosystems) was used to perform qRT-PCR in StepONE plus FAST real-time PCR machine. The samples were analyzed from three independent experiments with technical triplicates, out of which one representative experiment is shown. The primers used are listed in Supplementary Table 1.

### Immunoblotting

For immunoblotting, the whole-cell lysates (WCL) were made by lysing the cells in lysis buffer (20 mM Tris pH 8.0, 150 mM NaCl, 0.1% SDS,0.5% Sodium deoxycholate, 1% NP-40, 1 mM EDTA). Briefly, the cells were lysed in lysis buffer for 1 h in ice with intermittent vortexing. The lysates were electrophoresed on 15%, 11%, or 7.5% SDS-PAGE and transferred onto a nitrocellulose membrane. Primary antibodies were added to the blot overnight at 4 °C, before which the membrane was blocked with 5% non-fat dry milk in TBST. The antibodies used are listed in Supplementary Table 2. The immunoblots are quantified and normalized to loading control. The numerical reflect the band intensity in arbitrary units.

### Immunofluorescence

Immunofluorescence was perfume as described previously [16]. Briefly, 4% paraformaldehyde was used to fix the cells, then permeabilization with 1% Triton X100. 3% BSA was used to block the coverslips and incubated with the primary antibodies as indicated, followed by fluorophore-tagged secondary antibodies (Alexa 488 and 594) in PBST for 1 h each. The coverslips were mounted using a mounting medium containing DAPI and imaged using Zeiss LSM 800 Meta Confocal Microscope. The antibodies used are listed in Supplementary Table 2.

### Coimmunoprecipitation

Co-IP was performed using a previous protocol, as mentioned elsewhere [16]. Cross-linking of cells with 1% formaldehyde was followed by neutralization with 125 mM glycine. Then cells were harvested and lysed using RIPA lysis buffer (50 mM Tris pH 8, 150 mM NaCl, 0.1% SDS, 0.5% Sodium deoxycholate, 1% NP-40, 1 mM EDTA and supplemented with complete protease inhibitor cocktail). The cell lysate was sonicated, followed by centrifugation at 13000 rpm for 10 min. After pre-clearing with normal sheep serum, immunoprecipitation was set using respective antibodies overnight. After that, dynabeads blocked in 5% BSA were added to the lysate to pull the protein-antibody complex, and the drawn beads were washed three times with the sample buffer to remove the non-specific interactions. Finally, western blotting is done for the analysis of immunoprecipitation.

### ChIP and Re-ChIP assay

ChIP assays were performed as previously described [16]. Briefly, 1% formaldehyde was used to crosslink the cells for 10 min at RT, and 125 mM glycine for 10 min at RT was used to terminate the reaction. Firstly, the cell lysis buffer (5 mM PIPES pH 8.0, 0.5% NP-40, 85 mM KCl) with a protease inhibitor cocktail was used to resuspend the cells for cell lysis. The nuclei were isolated, followed by resuspension in nuclei lysis buffer (50 mM Tris-HCl pH 8.0, 1% SDS, 10 mM EDTA). Following this, the chromatin was sheared, and various antibodies were used for immunoprecipitation or IgG as a negative control. The immunoprecipitant was pulled down using Dyna beads followed by sequential washes with RIPA buffer, high salt (500 mM NaCl) buffer, LiCl wash buffer, and TE buffer. The beads were then subjected to RNase A digestion followed by Proteinase K treatment. The complex was then decrosslinked by heating at 65 °C overnight. Next, the phenol-chloroform extraction method was used to isolate the ChIP DNA, precipitated using ethanol. ChIP DNA was dissolved in water and analyzed using gene-specific primers via qPCR. Three independent experiments were performed with technical triplicates.

Re-ChIP assays were done as mentioned previously [42]. Briefly, the immunocomplex was pulled down with the first desired antibodies, treated with 0.05 M DTT, and incubated at 30 °C for 1 h. Then the sample was diluted 20-fold, and the immunoprecipitation reaction was set with a second antibody. The purified DNA was amplified by real-time PCR, and further analysis was done using specific primers. Finally, the fold-enrichment values were calculated after normalizing with the values obtained from the first pull-down with desired antibody and IgG.

The antibodies used are listed in Supplementary Table 2.

### Luciferase assay

Luciferase reporter assay was performed with cells seeded in a 12-well culture dish. A pGL3-MAPT-RARE promoter or pGL3-MAPT-IGRR or pGL3 basic empty vector was transfected in SK-N-SH or SH-SY5Y cells with Lipofectamine 2000 (Invitrogen) as per the manufacturer’s protocol. Post 6 h of transfection, the cells were replenished with a differentiation medium supplemented with or without ATRA for the next 24 h. pRL-CMV vector was co-transfected in an individual experiment for normalization. The cells were lysed in a passive lysis buffer with vigorous shaking the next day. The firefly/renilla luciferase activity was measured in the Luminometer using the Dual-Luciferase Reporter Assay System—(Promega, Catalog #E1910) as per the manufacturer’s protocol. The samples were analyzed from three independent experiments with technical triplicates, out of which one representative experiment is shown.

### Copy number determination

To determine the copy number of *MAPT* and *MAPT213*, a standard curve was generated using in vitro transcribed *GAPDH* mRNA. The in vitro transcribed *GAPDH* mRNA was diluted from 10^2^–10^9^ molecules and analyzed by real-time PCR. The Ct values obtained were plotted against the copy number. Copy number was calculated from nanograms using a standard formula. The Ct values for *MAPT* and *MAPT213* from all different experimental conditions (+/− ATRA in +/− ZMYND8) were determined from 25 ng total cellular RNA and were plotted to the standard curve. A standard curve was prepared to determine the molecule copy number. To calculate the copy number of *MAPT* and *MAPT213* per SK-N-SH cell, the total RNA obtained per SK-N-SH cell under all experimental conditions was determined by generating a standard curve of the number of SK-N-SH control or *ZMYND8*-KO cells versus total RNA. Cells were counted using a hemocytometer, and RNA was isolated by the TRIzol method and quantified by Nanodrop.

### Cell fractionation

The cells were lysed in cytoplasmic extraction buffer (20 mM HEPES pH 7.9, 0.075% NP-40, 10 mM KCl, 0.1 mM EDTA, 1X PIC,5 mM vanadyl ribonucleoside complex) and incubated on ice for 10 min, with intermittent vortexing. The cytoplasmic extract was collected after centrifugation at 3000 rpm for 5 min at 4 °C. Nuclear extraction buffer (20 mM HEPES pH 7.9, 1% NP-40, 500 mM NaCl, 25% Glycerol, 1 mM EDTA, 1X PIC, 5 mM vanadyl ribonucleoside complex) was added to the pellet followed by ice incubation for 10 min. The nuclear extract was collected after centrifugation at 13000 rpm at 4 °C for 10 min. Both cytoplasmic and nuclear extracts were divided equally. One was used for RNA isolation by the TRIzol method, and the other was used immunoblotting to confirm the fractionation. H3 was used as a nuclear marker, whereas Tubulin was a cytosolic marker.

### Nuclear run-on transcription

The nuclear run-on transcription was performed as previously described [43]. Briefly intact nucleus was isolated from either control or *ZMYND8*-KO (+/− ATRA) treated cells using NP-40 lysis buffer (10 mM Tris-HCl pH 7.4, 150 mM NaCl, 3 mM MgCl_2_, 0.5% NP-40). Immunoblotting with cytosolic or nuclear markers was used to verify the cellular fractionation. In nuclei storage buffer (50 mM Tris-HCl pH 8.3, 40% glycerol, 5 mM MgCl_2_, 0.1 mM EDTA) the isolated intact nuclei were resuspended. The run-on transcription was performed at 30 °C for 30 min in transcription buffer (10 mM Tris HCl pH 8.3, 2.5 mM MgCl_2_, 150 mM KCl, 2 mM DTT, 1 mM ATP, 1 mM UTP, 1 mM GTP, 1 mM CTP, 100U RNase inhibitor). TRIzol was used to isolate the total RNA and further subjected to DNaseI (New England Biolabs) treatment at 37 °C for 15 min. Inactivation of DNaseI was performed at 75 °C for 5 min. The isolated RNA was reverse transcribed. The absence of any genomic DNA was confirmed by using no reverse transcriptase control.

### Polysome isolation

Total polysome was isolated and fractionated using sucrose gradient fractionation as described elsewhere [43]. Briefly, cells were incubated with 100 μg/ml of cycloheximide (Sigma) at 37 °C for 10 min. The cells were lysed in polysome extraction buffer (10 mM HEPES pH 8.0, 5 mM MgCl_2_, 25 mM KCl, 1% Triton X100, 1 mM DTT, 1% sodium deoxycholate, 5 mM vanadyl ribonucleoside complex, 1X protease inhibitor cocktail (Roche) containing 100 μg/ml cycloheximide). The cytoplasmic extract was isolated, and total RNA was quantitated using NanoDrop. An equal amount of cytoplasmic lysate was fractionated on a sucrose gradient (10–50%) and ultracentrifuged at 190,000 g for 90 min at 4 °C in Sorvall WXUltra100 (Thermo Fischer Scientific) with AH650 rotor. Total RNA was isolated and analyzed from each volume 500 μl fraction and percent of RNA was calculated.

### RNA seq and analysis

#### Transcriptome assembly and differential expression

The library for total RNA-sequencing was prepared and sequenced by Novogene USA. Data were analyzed as described elsewhere [44], [45]. Briefly, Mapping NGS paired-end reads to the human transcriptome (GRCh38) were carried out. De-multiplexing and adapter trimming were performed. The quality of the data was assessed using the Trim_Galore! (Babraham Bioinformatics) package and low-quality reads were removed (Phred score < 25). Paired-end reads were mapped to the human genome (GRCh38) using the Hisat2 aligner. SAM files were converted into BAM files, then sorted and indexed using Samtools. Read counts were computed using the alignment files and the FeatureCounts package with GENCODE (v28) as reference annotation. Counts normalization and differential gene expression analyses were carried out using the DESeq2 R package. Briefly, input files for DESeq2 are metadata files with conditions and the read counts obtained from FeatureCounts, and two replicates were used for each condition. Differentially expressed genes were extracted by applying *p*-value (<0.05), base mean (>20) cutoffs, and a 1-fold change cutoff. The regions were annotated using the R package biomaRt annotation querying Ensembl database. Post-sequencing analyses partly were performed by Kinsight Bio Analytics LLC.

#### Heatmaps and gene ontology

Heatmaps were generated, and gene ontology analyses were performed as described elsewhere [44], [45]. Briefly, heatmaps were generated in the statistical software R (version 4.0.3) using the R package plotly. Biotype classification of the transcripts was performed using the Ensembl (REST API) and biomaRt R package on filtered differentially expressed genes. Gene Ontology (GO) was performed by querying the bioinformatic Database for Annotation, Visualization, and Integrated Discovery (DAVID).

### ENCODE data sets

We analyzed the ChIP-seq data from the ENCODE portal (https://www.encodeproject.org/) and visualized it on UCSC genome browser [29] with the following identifiers: ENCSR000FCS (H3K4me3; Lab: Peggy Farnham, USC), ENCSR000FCR (H3K4me1; Lab: Peggy Farnham, USC), ENCSR000FCU (H3K27ac; Lab: Peggy Farnham, USC) [46].

### Genomic data set availability

The previously reported data sets herein are ChIP-seq from 293 T cells (GSE51633) as reported in [47], and 293 cells (GSE81696) as reported in [19].

### Statistical analysis

ChIP-qPCR and qRT-PCR experiments were performed in three technical replicates. Each experiment was repeated three times at least. Along with the regular bar graph representation, Box Whisker plot analysis of all the ChIP-qPCR (Supplementary Fig. 6) and qRT-PCR (Supplementary Fig. 7) experiments were performed. Statistical difference was obtained by an unpaired two-tailed student *t*-test using the GraphPad Prism Software. One-way Anova was performed to determine the statistically significant difference. GraphPad Prism 8.0.2(GraphPad Software Inc., La Jolla, CA) was used for the analysis. *P*-values, *P ≤ 0.05; **P ≤ 0.001 were considered significant. The standard deviation (s.d.) of the mean (±SEM) was represented as error bars.

Since qPCR is log-transformed before analysis, which already helps with influential outliers or skewed data, we have used parametric tests. However, we have performed the Shapiro-Wilk test, which indicated that our data points passed the normality test (alpha = 0.05).

### Reporting summary

Further information on research design is available in the Nature Research Reporting Summary linked to this article.

## Supplementary information

Supplemental Material

Supplementary Table 3

Supplementary Table 4

Supplementary Table 5

Supplementary Table 6

Uncropped Blots

Reporting Summary_Springer Nature

## Data Availability

The RNA seq data is available from the NCBI’s Gene Expression Omnibus (GEO) database using the following accession number: GSE196029.
