# Supplementary material for: ZMYND8 suppresses MAPT213 LncRNA transcription to promote neuronal differentiation
Source: Cell Death Dis. 2022 Sep 5;13(9):766. doi: 10.1038/s41419-022-05212-x (PMC9445031; doi:10.1038/s41419-022-05212-x)
Supplement: Supplementary file 1 — Supplemental Material [file 41419_2022_5212_MOESM1_ESM.docx]

**ZMYND8 Suppresses *MAPT213* LncRNA Transcription To Promote Neuronal Differentiation**

**Adhikary *et al.* (2022)**

**Supplementary Information**

**Supplementary Figures**

**Supplementary Tables**

**Supplementary Figure 1**

**Supplementary Figure 1: ZMYND8 regulates differentiation and pluripotency genes.**

(A, B) qRT-PCR showing expression of *ZMYND8*, differentiation promoting genes *MAPT*, *TUBBIII*, *DRD2*, *SCN2A*, *LAMA1*, *NEUROG1* or pluripotency genes *REST*, *POU5F1*, *NANOG*, *SOX2* in *ZMYND8* knockdown (*ZMYND8*-sh1) or control NSCs with or without bFGF (A), *ZMYND8* knockout (ZMYND8-KO) or Control SK-N-SH cells (B) with or without ATRA treatment. (C) Cell morphology of Control or *ZMYND8* knockout (*ZMYND8*-KO) SK-N-SH cells with or without ATRA treatment. Scale bar indicates 20μm.

(D) Immunoblot (E) and qRT-PCR (E) showing expression of ZMYND8, differentiation promoting genes or pluripotency genes in *ZMYND8* knockdown (*ZMYND8*-sh1) or control SH-SY5Y cells with or without ATRA treatment. The immunoblots are quantified and represented as numerical reflecting the band intensity normalized to loading control.

Error bars indicate sem.; n=3; three independent experiments. A two-tailed t-test was used to calculate *P*-values. **P*<0.05; ***P*<0.01.

**Supplementary Figure 2**

**Supplementary Figure 2: ZMYND8 co-localizes with other epigenetic regulator proteins at the TSS.** Heat maps of FLAG-ZMYND8, H3K4me3, p300, H3K27ac, BRD4 and H3K4me1 (in HEK293 cell line) represented by reanalysis of available ChIP-Seq data (GSE51633 and GSE81696).

**Supplementary Figure 3**

**Supplementary Figure 3: Absence of ZMYND8 shows increased transcriptional activity from intra-regulatory regions of neuronal genes.**

(A) Bar plots of qPCR showing enrichment of H3K27ac at the *MAPT* intra-gene regulatory region (IGRR) in *ZMYND8*-knockdown (*ZMYND8*-sh1) or control SH-SY5Y cells with or without ATRA treatment.

(B, C) Bar plots of qPCR showing enrichment of different domains of ZMYND8 (B) and H3K4me1 (C) at *MAPT* promoter or intra-gene regulatory region (IGRR) in *ZMYND8*-knockout (ZMYND8-KO) or control SK-N-SH cells with or without ATRA treatment.

(D, F) UCSC Genome Browser track showing the histone modifications at the *DRD2* (C) and *SCN2A* (E) genes data was accessed from ENCODE.

(E, G) Bar plots of qPCR showing enrichment of H3K27ac and H3K4me3 at the *DRD2* (D) and *SCN2A* (F) intra-gene regulatory region (IGRR) in *ZMYND8*-knockout (*ZMYND8*-KO) or control SK-N-SH cells with or without ATRA treatment.

(H, I) Luciferase assay measuring the firefly luciferase activity of *MAPT* promoter (H) or IGRR (I) in SH-SY5Y cells upon *ZMYND8*-knockdown (*ZMYND8*-sh1) with or without ATRA treatment. All firefly luciferase activities were normalized to renilla luciferase activity.

Error bars indicate sem.; n=3; three independent experiments. A two-tailed t-test was used to calculate *P*-values. **P*<0.05; ***P*<0.01.

**Supplementary Figure 4**

**Supplementary Figure 4: Association of ZMYND8 with RNA Polymerase II machinery at the promoter and IGRR of *MAPT* in SH-SY5Y cells.**

(A-E) Bar plots of qPCR showing enrichment of ZMYND8 (A), BRD4 (B), CDK9 (C), RNA pol II phospho S5 (D) and RNA pol II phospho S2 (E) at the *MAPT* promoter and IGRR in *ZMYND8* knockdown (*ZMYND8*-sh1) or control SH-SY5Y cells with or without ATRA treatment.

Error bars indicate sem.; n=3; three independent experiments. A two-tailed t-test was used to calculate *P*-values. **P*<0.05; ***P*<0.01.

**Supplementary Figure 5**

**Supplementary Figure 5: Characterization of *MAPT Intra gene transcript (MAPT213)*.**

(A-B) qRT-PCR showing expression of *MAPT* transcripts from promoter, 5’UTR, Exons 2-5 and 3’UTR (A) or *MAPT213*(B) in *ZMYND8* knockdown (*ZMYND8*-sh1) or control SH-SY5Y cells with or without ATRA treatment.

(C) Immunoblot showing the efficiency of cell fractionation. Tubulin and Histone H3 are used as cytosolic or nuclear marker, respectively.

(D) qRT-PCR showing expression of *MAPT Intra gene transcript* in nuclear and cytosolic compartments of *ZMYND8*-knockout (*ZMYND8*-KO) or control SK-N-SH cells with or without ATRA treatment. Nuclear pool was normalized to *U6 snRNA* and cytosolic pool to *18S rRNA*. Error bars indicate sem.; n=3; three independent experiments. A two-tailed t-test was used to calculate *P*-values. **P*<0.05; ***P*<0.01.

(E) Correlation of *ZMYND8* and *MAPT213* transcript expression in brain tissues using GTEx ^1^.

(F) DNA sequence conservation of *MAPT* and *MAPT213* between human and mouse. Percentage identity for the *MAPT* (Ref Seq) gene was obtained from the NIH/NCBI website by querying the HomoloGene database and obtaining the Pairwise Alignment Scores. On the other hand, the *MAPT 213* transcript was obtained from the Ensembl database by querying the 213 transcript and carrying out a BLASTN against mouse GRCm39 (Genomic Sequence). Results from this query returned a total of 2186 his found, but only one hit with the overlapping gene *MAPT*. The region was 78bp in length (from the 5015bp total) and a percentage identity of 85.9%.

**Supplementary Figure 6**

**Supplementary Figure 6: (A-P) Box Whisker plots for the ChIP experiments represented in Figures 4-6.** A-C panels represents the ChIPs of Figure 4, D-H panels that of Figure 5 and I-P panels that of Figure 6. One-way ANOVA was used to compute statistically significant differences. Boxes marked with a, b, c are statistically significant from each other padj-Value=<0.05.

**Supplementary Figure 7**

**Supplementary Figure 7: (A-J) Box Whisker plots for the qRT-PCR experiments represented in Figures 1, 7 and 8.** The panel A represents the qRT-PCR of Figure 1B, the panels B-C that of Figure 6 B and D and panels D-H that of Figure 6E-L. I-J panels that of Figure 8 A and B. Statistical analysis has been performed by Student’s t test.

**Supplementary Table S1: List of qRT-PCR Primers**

| Gene | Forward 5’-3’ | Reverse 5’-3’ |
| --- | --- | --- |
| *ZMYND8* | CAGAAAATGAAACAGCCAGGG | ACTTTGCATCAGCCAGGAAG |
| *MAPT* | GCGGCAGTGTGCAAATAGTCTACAA | GGAAGGTCAGCTTGTGGGTTTCAAT |
| *TUBBIII* | GCCTCAAGATGTCCTCCACC | CGTACATCTCGCCCTCTTCC |
| *DRD2* | TGCAGACCACCACCAACTACCTGAT | GAGCTGTAGCGCGTATTGTACAGCAT |
| *LAMA1* | GGAGTTAAGAACACGGAGTC AG | GTCTTGTGTAGTTCTCGTAGCA G |
| *SCN2A* | GCGAGTCAGATATGGAGGAAAG | AACAGGCTTCAGGTTCAAGG |
| *NEUROG1* | GCCTCCGAAGACTTCACCTACC | GGAAAGTAACAGTGTCTACAAAGG |
| *POUSF1* | GAAGGAGAAGCTGGAGCAAA | CCACATCGGCCTGTGTATATC |
| *NANOG* | TCCTGAACCTCAGCTACAAAC | GCGTCACACCATTGCTATTC |
| *SOX2* | GCCCACCTACAGCATGTCCTA | TGGGAGGAAGAGGTAACCACAG |
| *REST* | CCTTTCGCTGTAAGCCATGC | TGGTGTTTCAGGTGTGCTGT |
| *GFAP* | TCCTGGAACAGCAAAACAAG | CAGCCTCAGGTTGGTTTCAT |
| *U6 snRNA* | CTCGCTTCGGCAGCACATATACT | ACGCTTCACGAATTTGCGTGTC |
| *18S rRNA* | GATTCCGTGGGTGGTGGTGC | AAGAAGTTGGGGGACGCCGA |
| *GAPDH* | AATCCCATCACCATCTTCCAG | ATGACCCTTTTGGCTCCC |
| *MAPT ncRNA* | CTAACCCTCACCATCCTTCTAC | GTTGTGGAGGCTGTGTCTAAG |
| *MAPT Promoter* | GTAGTCCCAGCAACTCAGAAG | TGGCACAATCGTAGCTCAC |
| *MAPT 5’UTR* | CCACAGCCACCTTCTCC | TCCTGGTTCAAAGTTCACCTG |
| *MAPT Exon2* | ATGCACCAAGACCAAGAGG | TTAGCATCAGAGGTTTCAGAGC |
| *MAPT Exon3* | ATGCACCAAGACCAAGAGG | TGTCACATCTTCCGCTGTTG |
| *MAPT Exon4* | ATGTGACAGCACCCTTAGTG | TGTCTCCAATGCCTGCTT |
| *MAPT Exon5* | ATGTGACAGCACCCTTAGTG | GCAGCTTCGTCTTCCAGG |
| *MAPT 3’UTR* | CAATCACTGCCTATACCCCTC | TGCCTTCCCTTAATTTCACCC |
| *MAPT-213 (cloning primers)* | CGGGGTACCAGAGCCTCATCCTTGAACG | TCGATGATATCAAACAGAGTCTCACTCACTC |

**Supplementary Table 2: List of Antibodies**

| **Antibodies** | **Company** | **Cat No.** |
| --- | --- | --- |
| H3 | Abcam | ab1791 |
| H4 | Abcam | ab10158 |
| H2B | Abcam | ab1790 |
| H3K4me3 | Abcam | ab8580 |
| H3K27me3 | Abcam | ab6002 |
| H3K36me2 | Abcam | ab9049 |
| H4K16ac | Abcam | ab109463 |
| H3K27ac | Abcam | ab4729 |
| H4K5ac | Abcam | ab51997 |
| H4K8ac | Abcam | ab15823 |
| BRD4 | Abcam | ab128874 |
| CDK9 | CST | 2316 |
| RNA Pol II phospho S5 | Abcam | ab5131 |
| RNA Pol II phospho S2 | Abcam | ab5095 |
| Non-phospho CTD | Santa Cruz | sc-899 |
| ZMYND8 | Sigma | HPA020949 |
| KDM5C | Abcam | ab34718 |
| EZH2 | Abcam | ab191250 |
| FLAG | Sigma, Abcam | A8592, ab1162 |
| NESTIN | Abcam | ab22035 |
| SOX2 | Abcam | ab97959 |
| GFAP | Abcam | ab7260 |
| TAU Nterm | Santa Cruz | sc166060 |
| TAU Cterm | Abcam | ab64193 |
| TAU 5 | Abcam | ab80579 |
| MAP2 | Abcam | ab5392 |
| β-III TUBULIN | Abcam | ab18207 |
| REST | Millipore | 07-579 |
| Oct4 | Santa Cruz | sc5279 |
| CHD4 | Abcam | ab70469 |
| MTA1 | Bethyl | A300-280A |
| HDAC1 | Abcam | ab7028 |
| CBP | Abcam | ab2832 |
| p300 | Abcam | ab14984 |
| GAPDH | Abcam | ab8245 |
| Rabbit IgG HRP | Sigma | A1949 |
| Mouse IgG HRP | Promega | W402B |
| Rabbit Alexa Fluor-488 | Invitrogen | A11034 |
| Rabbit Alexa Fluor-564 | Invitrogen | A11037 |
| Mouse Alexa Fluor-564 | Invitrogen | A11032 |
| Mouse Alexa Fluor-488 | Invitrogen | A28175 |

**Reference**

1 Consortium, G. T. The GTEx Consortium atlas of genetic regulatory effects across human tissues. *Science* **369**, 1318-1330, doi:10.1126/science.aaz1776 (2020).
