# Supplementary material for: ZMYND8 suppresses MAPT213 LncRNA transcription to promote neuronal differentiation
Source: Cell Death Dis. 2022 Sep 5;13(9):766. doi: 10.1038/s41419-022-05212-x (PMC9445031; doi:10.1038/s41419-022-05212-x)
Supplement: Supplementary file 6 — Uncropped Blots [file 41419_2022_5212_MOESM6_ESM.pptx]

## Slide 1
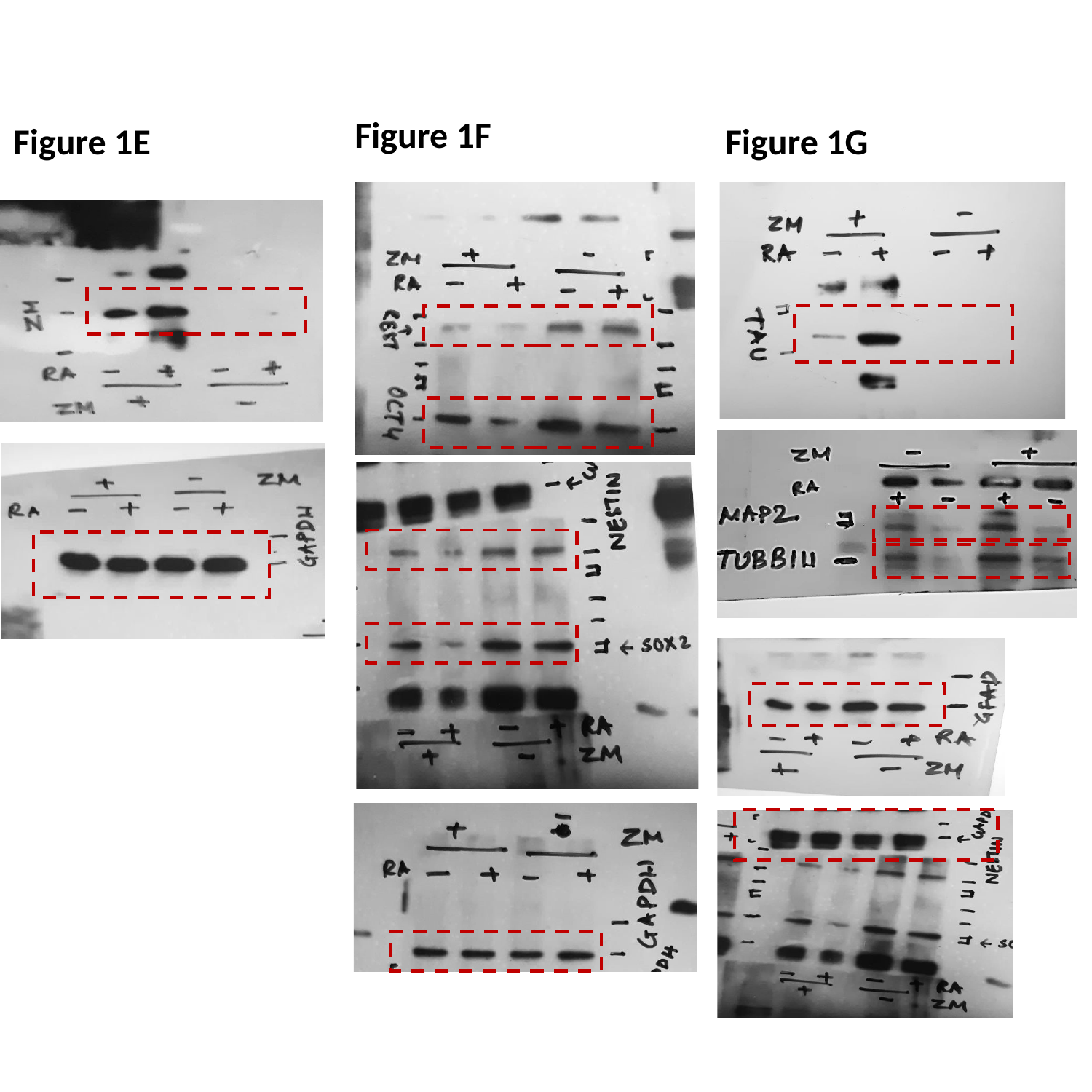

Figure 1F
Figure 1G
Figure 1E

## Slide 2
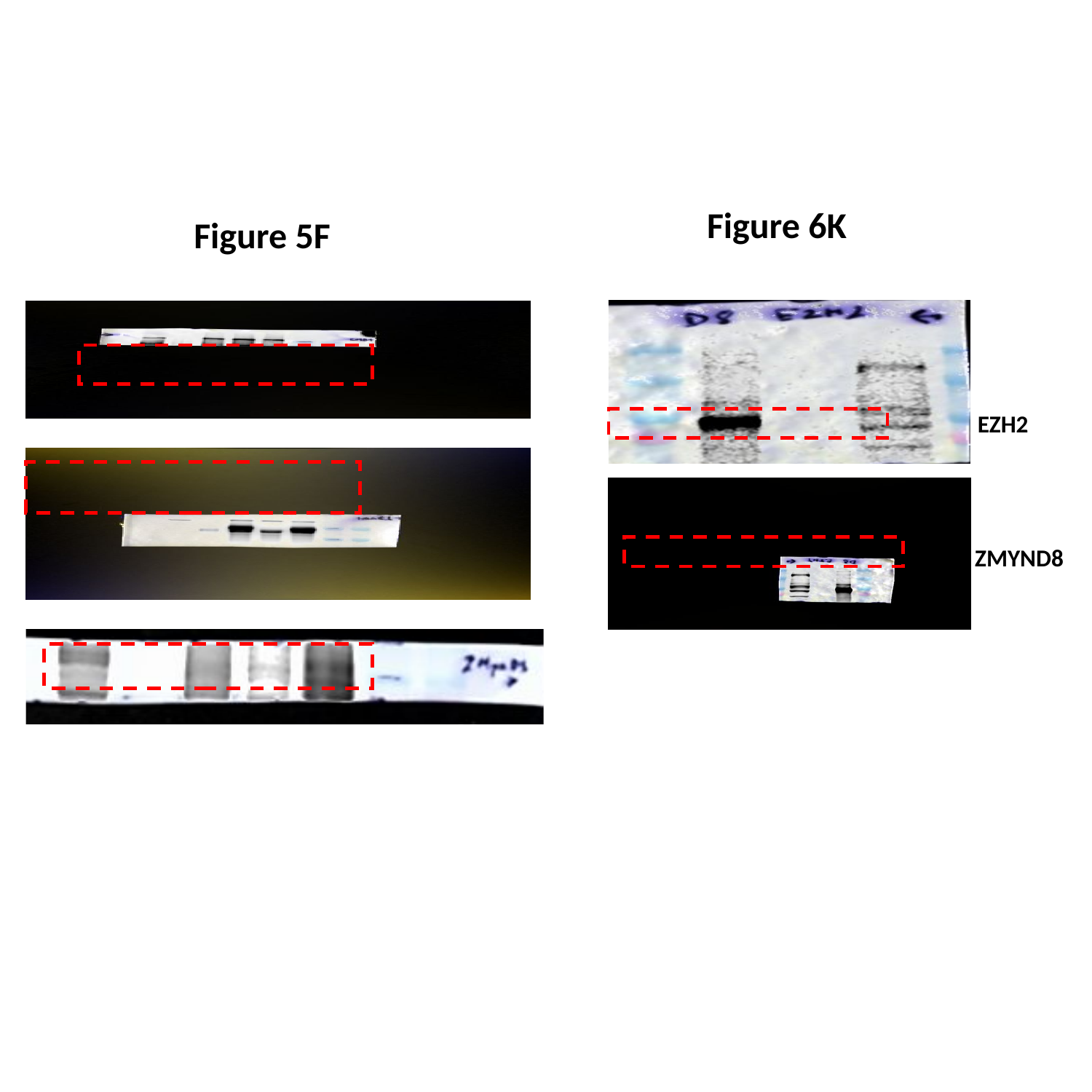

Figure 6K
EZH2
ZMYND8
Figure 5F

## Slide 3
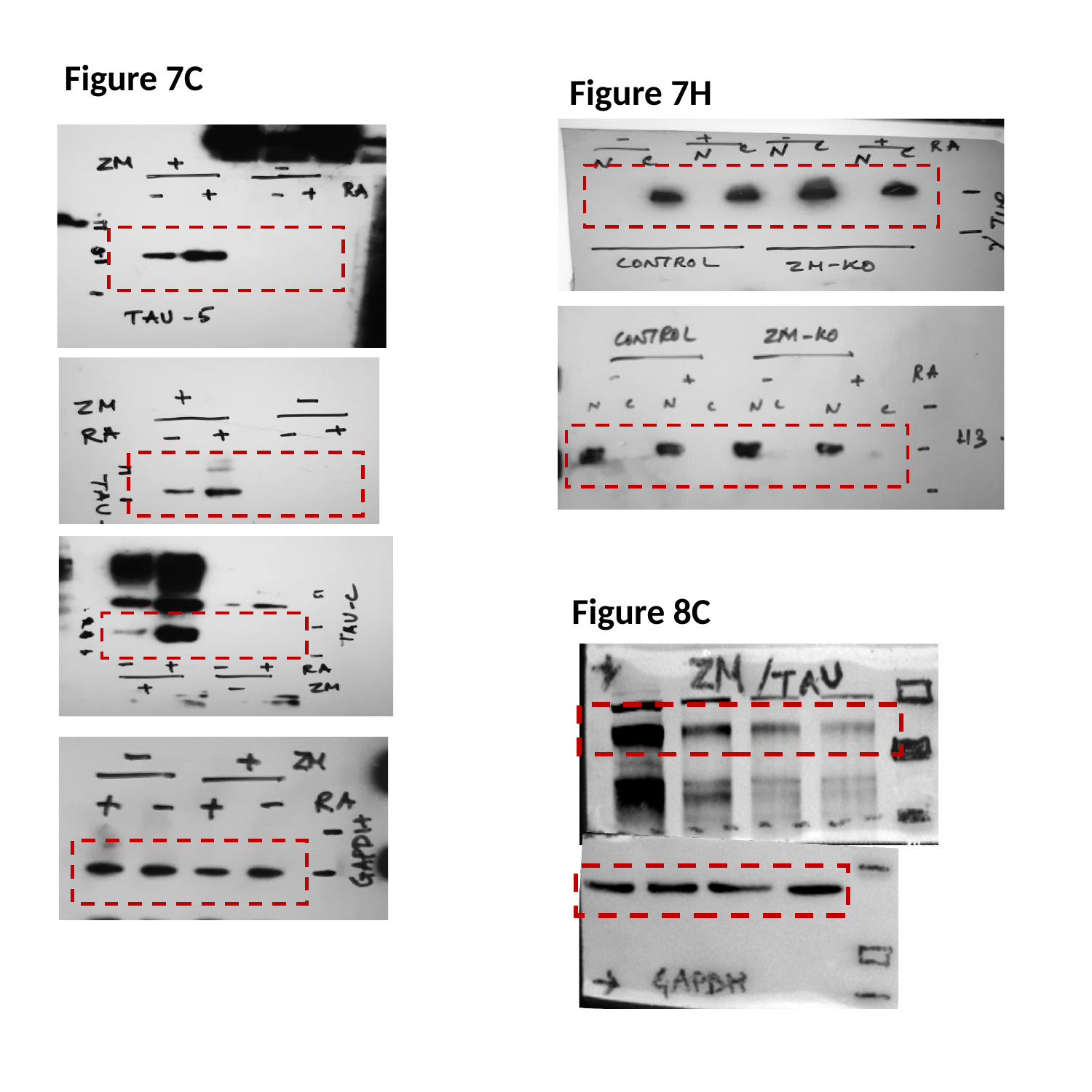

Figure 7C
Figure 7H
Figure 8C

## Slide 4
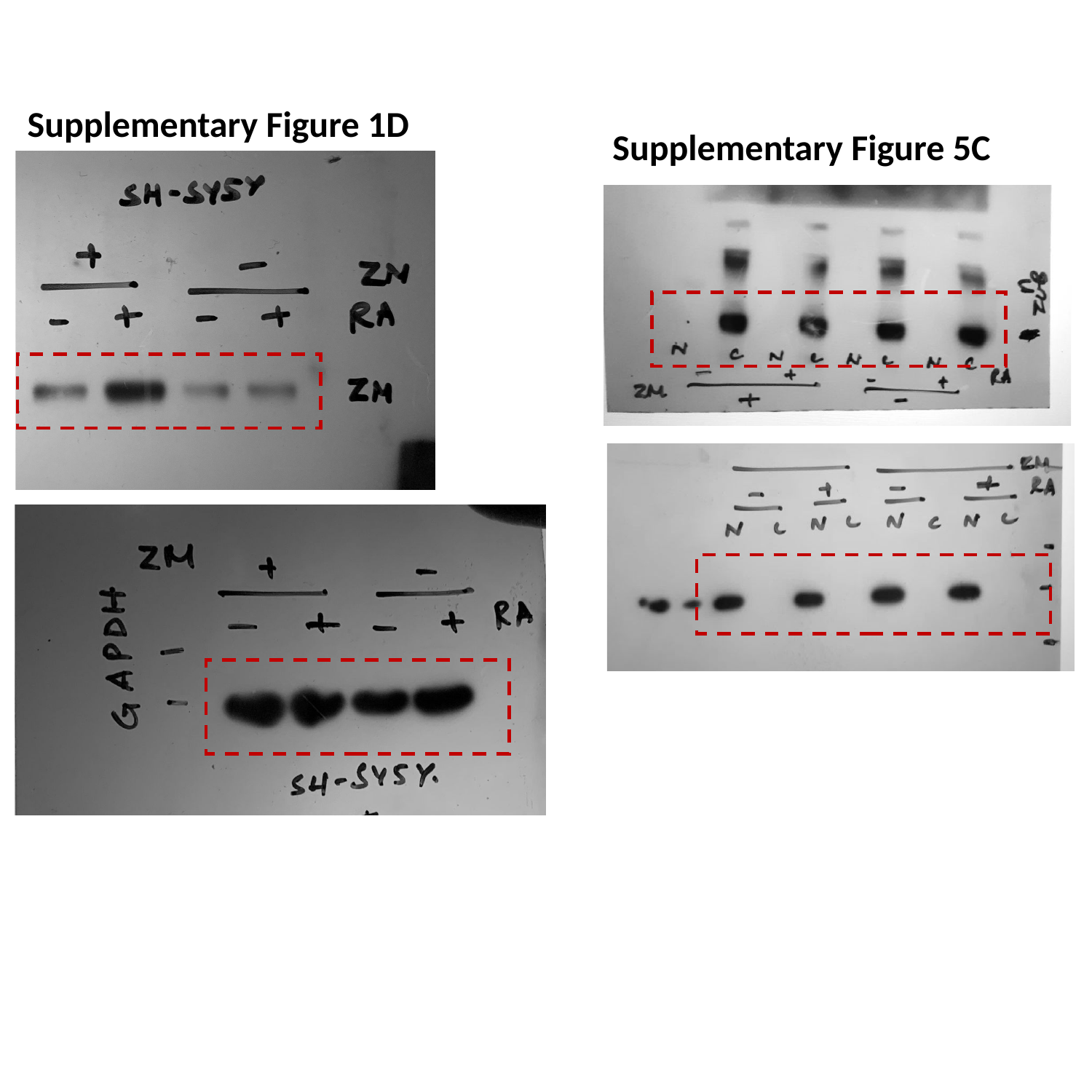

Supplementary Figure 1D
Supplementary Figure 5C
